# Therapeutic potential of a novel combination of Curcumin with Sulfamethoxazole against carbon tetrachloride-induced acute liver injury in Swiss albino mice

**DOI:** 10.1186/s43141-020-00027-9

**Published:** 2020-05-04

**Authors:** Rasha Fekry Zahran, Zeinab M. Geba, Ashraf A. Tabll, Mohammad M. Mashaly

**Affiliations:** 1 Department of Chemistry (Biochemistry division), Faculty of Science, Damietta University, New Damietta, Egypt; 2 Department of Microbial Biotechnology, Division of Genetic Engineering and Biotechnology, National Research Centre, Cairo, 12622 Egypt; 3 Department of Chemistry, Faculty of Science, Damietta University, New Damietta, Egypt

**Keywords:** Fibrosis, Carbon tetrachloride, Curcumin, Sulfamethoxazole, Oxidative stress, Histopathology

## Abstract

**Background:**

In the current study, we have investigated the effect of each of curcumin (CUR) and sulfamethoxazole (SMX) either separate or mixed together (CUR + SMX) on biochemical, hematological and histological alternations associated with carbon tetrachloride (CCl_4_)-induced liver fibrosis in mice.

**Results:**

CCl_4,_ caused changes of several biomarkers, proving its hepatotoxic effects, such as an increase in aminotransferases liver enzymes alanine and aspartate transaminases (ALT, AST), malondialdehyde (MDA), and nitric oxide (NO) formation, with a decrease in superoxide dismutase (SOD), glutathione reductase (GSSG), total antioxidant capacity (TAO), glutathione (GSH), total protein, and albumin, compared to a negative control mice group. Compared to the CCl_4_ group of mice, the CUR and SMX separate and/or together (CUR + SMX) treatments showed significance in (*p* < 0.001), ameliorated liver injury (characterized by an elevation of (ALT, AST) and a decrease (*p* < 0.001) in serum albumin and total protein), antioxidant (characterized by a decrease in (*p* < 0.001) MDA, NO; an increase (*p* < 0.001) SOD, GSSG, TAO; and reducing GSH), hematological changes (characterized by a decrease (*p* < 0.001) in white blood cells count and an increase (*p* < 0.001) in platelets count, hematocrit levels, hemoglobin concentration, and (*p* < 0.05) red blood cells count), SDS-PAGE electrophoresis with a decrease in protein synthesis and changes in histological examinations.

**Conclusions:**

CUR and SMX either separate or together (SUR + SMX) may be considered promising candidates in the prevention and treatment of liver fibrosis.

## Impact statement

Liver diseases particularly liver fibrosis, cirrhosis, and hepatocellular carcinoma are major health problems worldwide and in Egypt. The current work reports on the impact of the therapeutic potential of curcumin (CUR) and/or sulfamethoxazole (SMX) against carbon tetrachloride-induced acute liver injury in Swiss albino mice. We demonstrated the CUR and SMX used separate or mixed together may be promising therapy in the prevention and treatment of liver fibrosis.

## Background

**Fig. 1:**
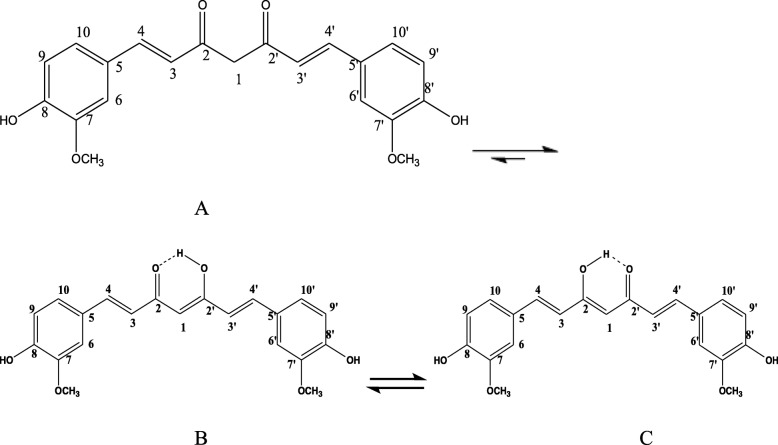
Keto (**a**) and enol (**b**, **c**) forms of curcumin, CUR

The liver carries out an essential job in the control of various physiological processes and it controls some vital functions, for example secretion, storage, and metabolic activities. It is frequently exposed to different xenobiotic and therapeutic agents. It detoxifies exogenous (toxic compounds) and endogenous (waste metabolites) substances of organisms and integrates helpful operators [1]. As far as anyone is concerned, no reports are available regarding actual curative therapy agents for liver disorders or diseases and most of the existing remedies only aid in healing or regeneration of liver [2]. Fibrosis, a general clinical condition, can be seen in various organs, but mostly in the liver. It is regularly connected with the end phase's of chronic inflammatory diseases [3, 4]. Liver fibrosis is the phenomena of the advancementof a hepatic scar because of chronic liver injury, coming about because of complex interconnected changes in cell population, extracellular matrix (ECM), and cytokines [5, 6]. For this study, fibrosis utilized carbon tetrachloride (CCl_4_)-induced animal model [7, 8], published reports [9], showed CCl_4_ as xenobiotic to induce acute and chronic tissue injuries through bio-enactment of the stage I cytochrome P450 enzymes to deliver receptive metabolic trichloromethyl radicals (CCl_3_) and peroxy trichloromethyl radicals (OOCCl_3_). These receptive metabolites have potential to covalently bond with enormous particles, for instance, lipids, proteins, and nucleic acids, pushing them out of their typical crucial usefulness. CCl_4_ injection lead to an expansion type of free radicals that are chemically highly reactive, and if they do not cause injury or necrosis, they bring about genuine unsafe impacts, such as hepatic fibrosis, copying the oxidative stress that has a fibrogenic effect on hepatic stellate cells (HSC) [3, 5, 6, 10, 11]. Curcumin, CUR (Fig. 1): 1, 7-bis (4-hydroxy-3-methoxyphenyl) hepta-1, 6-diene-3, 5-dione. (C_21_H_20_O_6_, molecular weight = 368.38) is the most bioactive structure among the curcuminoid chemical compounds that are extracted from the powdered dry rhizome of *Curcuma longa Linn* (*Zingiberaceae*), which is a perennial herb widely cultivated in the tropical region of Asia, especially in India [12, 13]. The medicinal value of curcumin has been well recognized. It has been proven that curcumin has anti-cancer [14], chemo preventive effect [15], antioxidant [16], antiproliferative [17], immunomodulatory [18], hepatoprotective [19], and anti-inflammatory properties [20]. Furthermore, CUR has shown to inhibit fibrosis; however, the specific mechanism of curcumin bioactivity still remains unclear [21].

**Fig. 2:**
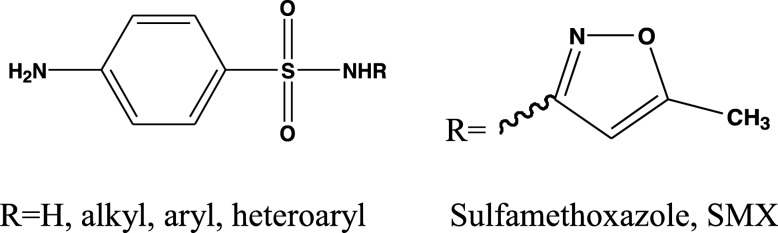
General structure of sulfonamides

In contrast, the sulfonamides are a chemical family of synthetic organic compounds containing the sulfonamide (-SO_2_NH_2_) or substituted sulfonamide functional group [22, 23]. There are many recent reports on the biochemical investigation of many sulfonamides as carbonic anhydrase inhibitors [24], antibacterial and anti-biofilm [25], antifungal [26], anticancer [27], and anti-inflammatory active agents [28]. Nowadays, many sulfonamide-containing agents are in clinical use, under different trade names, in different countries. Some examples of these clinically applied agents, mentioned above are, the tyrosine-kinase inhibitor drug [29], anti-inflammatory drug [30], anti-psychotic drug [31], anti-cancer drug [32], anti-inflammatory drug [33], carbonic anhydrase inhibitor drug [34], anti-diabetes [35], antiarrhythmic [36], anti-glaucoma, antidiuretics, antiepileptics [37], sweeten products [38], and the antibiotic [39]. Moreover, the sulfa drug, sulfamethoxazole (SMX), (Fig. 2) is a well-known antibiotic, acting as a bacteriostatic agent against gram-positive and gram-negative bacteria [40]. It, also, has been reported that low-dose of trimethoprim-sulfamethoxazole combination could be a treatment of pneumocystis pneumonia in nonhuman immunodeficiency virus-infected patients, with a low rate of adverse reactions [41].

In this study, we have investigated the effect of each of curcumin (CUR) and sulfamethoxazole (SMX) either separate or together (CUR + SMX) on induced liver fibrosis of carbon tetrachloride (CCl_4_), and induced acute liver injury in Swiss albino mice. The study, also, included an investigation of the effects of these, previously mentioned, compounds on biochemical, hematological, and histological alternations associated with carbon tetrachloride (CCl_4_)-induced liver fibrosis in mice.

## Methods

### Chemicals

All reagents and solvents were of high quality and were purchased from commercial sources and were used as received. CCl_4_ was purchased from BDH Chemicals, Ltd., Poole, England. CCl_4_ was diluted 1:1 with Virgin Olive Oil. SMX, Boric acid powder, and Vanillin were purchased from El-Gomhouria Company for Drugs and Chemicals, Egypt. Dimethyl sulfoxide (DMSO), Ethanol, ≥ 99.8% (GC), n-Butyl amine, 99.5% and Ethyl acetate, ≥ 99.5% (GC) were purchased from Sigma-Aldrich Chemical Co., (St Louis, MO, USA). Acetylacetone (2, 4-pentanedione), 99+%, Triisopropyl borate, 98+%, were purchased from ACROS ORGANICS, (Geel, Belgium). Hydrochloric acid, 37%, extra pure, and sodium sulfate, anhydrous, 99+%, extra pure were purchased from Fisher Scientific UK. Kits used for the experiments of biochemical studies were purchased from Biodiagnostic Company, Dokki, Giza, Egypt.

### Preparation of curcumin (CUR)

Curcumin, CUR, was prepared according to reported modified methods [42–44].

### Analysis of curcumin (CUR)

The melting point (m.p.) of CUR was measured in an open capillary glass tube on a Gallenkamp melting point apparatus (Gallenkamp and CO, UK) and was uncorrected. Infrared (IR) spectrums of CUR was obtained on Perkin Elmer 1430 spectrophotometer with potassium bromide (KBr) disc, in the wavenumber range of 4000–400 cm^−1^. Its ^1^H nuclear magnetic resonance (NMR) (500 MHz, DMSO-d_6_) and ^13^C NMR (125 MHz, DMSO-d_6_) spectra were determined on JEOL’s NMR spectrometer (500 MHz, Japan), using DMSO-d_6_ as solvent. Chemical shifts are expressed as δ values (ppm), using tetramethylsilane (TMS) as an internal standard. The following abbreviations were used to indicate the NMR-signals: s (singlet), d (doublet), and br (broad). Electron impact (EI) mass spectral (MS) analysis at 70 eV of curcumin was performed on Thermo Scientific Trace DSQ II GC-MS (50–400 m/z), (Waltham, MA) system.

Measuring the melting point and the IR analysis of curcumin were performed at the Chemistry Department, Faculty of Science, Egypt, while its ^1^H and ^13^C NMR and MS spectral analysis were performed at the Spectral Analysis Unit, Chemistry Department, Faculty of Science, Egypt.

### Curcumin (1, 7-bis (4-hydroxy-3-methoxyphenyl) hepta-1, 6-diene-3,5-dione) (Fig. 1)

**Fig. 3:**
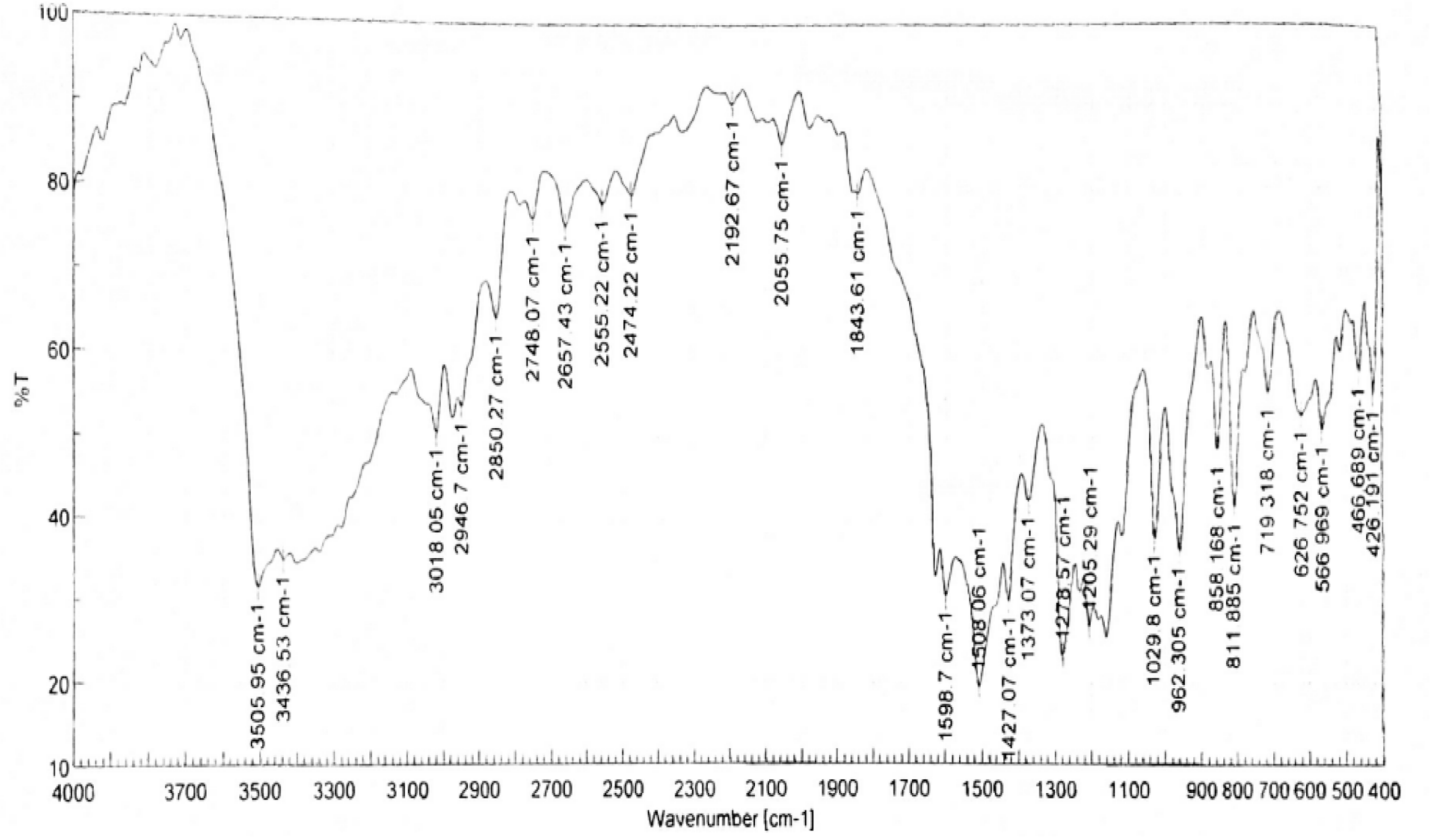
Infrared spectrum of curcumin

**Fig. 4:**
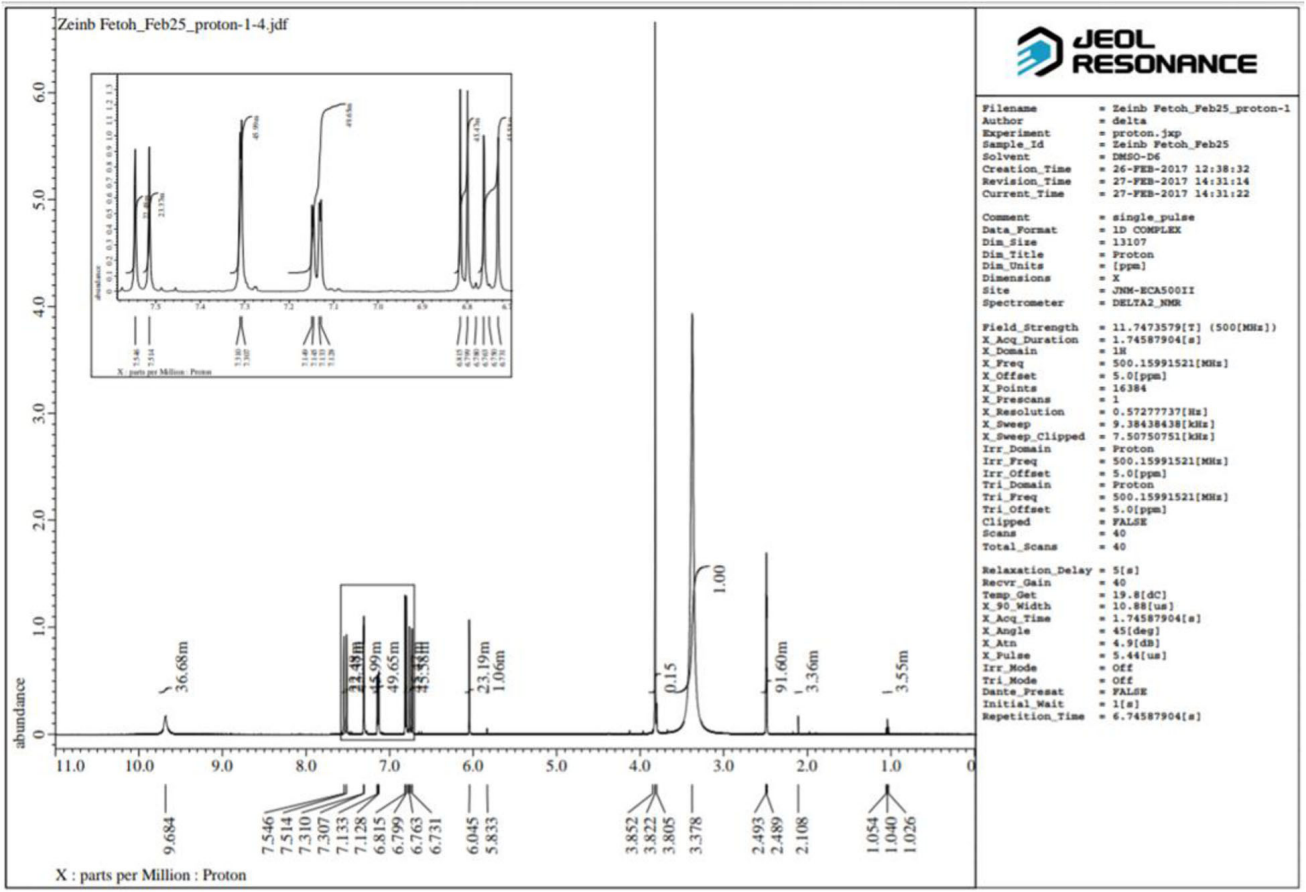
^1^H NMR (500 MHz) spectrum of curcumin in d_6_-DMSO

**Fig. 5:**
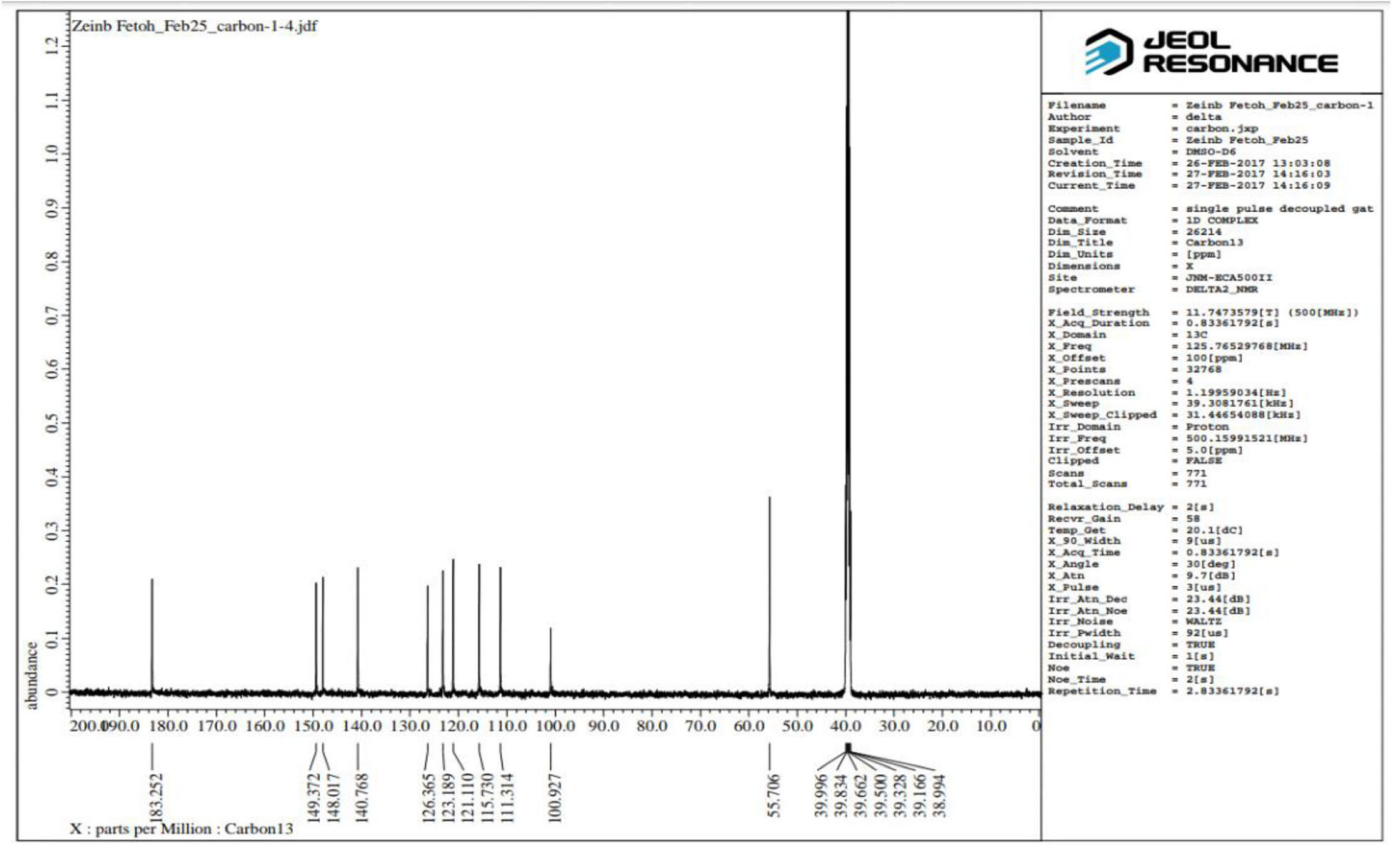
^13^C NMR (125MHz) spectrum of curcumin in d_6_-DMSO

It was obtained as a yellow-orange crystalline solid; m.p. 180–181 °C; IR (KBR, cm^−1^) (Fig. 3): 3505.95, 3436.53 (OH, H–bonded), 3018.05 (C–H aromatic stretching vibration), 2946.70, 2850.27 (CH_2_ and CH_3_ asymmetric stretching), 1643.61 (C=O), 1598.70 (C=C conjugated with C=O), 1508.06 (benzene ring), 1427.07 (enol C–O), 1373.07 (CH_3_ bending), 1278.57 (Phenolic C–O), 1029.80 (C–O–C in OCH_3_), 858.17, 811.89 (two adjacent aromatic C–H), 719.32 (C–H vibration aromatic); ^1^H NMR (DMSO-d_6_, 500MHz) (Fig. 4): δ (ppm) = 2.11(s, 2H, H-1, keto-form), 6.05 (s, 1H, H-1, enol-form), 6.75(d, 2H, H-3, H-3ˊ), 7.53 (d, 2H, H-4, H-4ˊ), 7.31(d, 2H, H-6, H-6ˊ), 9.68(s, br, 2H, Ar-OH), 6.80 (d, 2H, H-9, H-9ˊ), 7.13(dd, 2H, H-10, H-10ˊ), 3.83(s, 6H, OCH_3_); ^13^C NMR (DMSO-d_6_, 125MHz) (Fig. 5): δ (ppm) = 100.93( C-1), 183.25(C-1, C-1ˊ), 121.11 (C-3, C-3ˊ), 140.77(C-4, C-4ˊ), 126.37 (C-5, C-5ˊ), 111.31(C-6, C-6ˊ), 148.02( C-7, C-7ˊ), 149.37 (C-8, C-8ˊ), 115.73(C-9, C-9ˊ), 123.19(C-10, C-10ˊ), 55.71(OCH_3_); MS: m/z = 368 (0.92%)

### Ethics approval and consent to participate

All animal experiments conformed to the British Home Office Regulations (Animal Scientific Procedures Act 1986) and associated guidelines, EU Directive 2010/63/EU for animal experiments. This work launched after attaining permission from the scientific and ethical committees of Faculty of Science.

### Experimental animals

Adult male Swiss albino mice (total number, *n* = 180) were purchased from Zoology Department, Faculty of Science, Egypt, with average weight (25–30 gm each) and housed at the experimental animal house of the Faculty of Science. The animals were maintained in a properly controlled environment of temperature, humidity, and light. The mice were fed with autoclaved chow and filtered water and were adapted for a week prior to experimentation.

### Experimental design

The Swiss male adult albino mice were divided into nine (I–IX) groups (20 mice/each) as shown in Table 1. At the end of the experiment, mice fasted for 12 h and then blood samples were taken from the heart puncture under light ether anesthesia. Blood samples were collected for biochemical and hematological analysis. Animals were sacrificed and the liver dissected out and washed with isotonic saline and divided into two parts, the first part was stored at – 20 °C until assay for estimation of antioxidant parameters. The second part of the liver tissue was fixed in (10%) formalin for histopathology assessment.

**Table 1:** Experimental design

Groups	Dose	Method of section	Duration
Group I negative control	0.5 ml olive oil/kg b.w.	i.p	3 times a week for 5 weeks
Group II DMSO control	(0.2%) DMSO	i.p	3 times a week for 4 weeks
Group III CCl_4_ control	1 ml (50% in olive oil)/kg b.w.	i.p	3 times a week for 5 weeks
Group IV CUR group	CCl_4_ as group III followed by CUR 100 mg/kg b.w. in olive oil [45, 46]	CCl_4_ i.p CUR orally through a gastric tube	CCl_4_ 3 times a week for 5 weeks then 4 times a week for 4 weeks
Group V CUR control	100 mg/kg b.w. in olive oil [45, 46]	orally through a gastric tube	4 times a week for 4 weeks
Group VI SMX Group	CCl_4_ as group III followed by 15 mg SMX/kg b.w. in DMSO [47]	i.p	CCl_4_ 3 times a week for 5 weeks then 4 times a week for 4 weeks
Group VII SMX control	15 mg SMX/kg b.w. in DMS O[47]	i.p	4 times a week for 4 weeks
Group VIII Combination (CUR + SMX) group	CCl_4_ as group III followed by CUR as group V and SMX as group VII	CCl_4_ and SMX i.p CUR orally through a gastric tube.	CCl_4_ 3 times a week for 5 weeks then 4 times a week for 4 weeks
Group IX Combination (CUR. + SMX.) control	CUR as group V and SMX as group VII	CUR orally through a gastric tube. SMX i.p	4 times a week for 4 weeks

*CCl*_*4*_ carbon tetrachloride, *CUR* curcumin, *SMX* sulfamethoxazole, *i.p* intraperitoneally

### Biochemical analysis

Estimation of nitric oxide (NO) level was determined in liver tissues by using Biodiagnostic kit, Egypt (cat. no. NO2533), according to the method of Montgomery and Dymock [48]. Glutathione reductase (GSSG) was determined by using Biodiagnostic kit, Egypt (cat. no. GR2523), according to the method of Goldberg and Spooner [49]. The serum samples were collected for liver function tests. The activities of aspartate transaminase (AST) and alanine transaminase (ALT) were estimated by using Biodiagnostic kits, Egypt (cat. no. AS1061 (45), cat. no. AL1031 (45), according to the method of Reitman and Frankel [50]. Serum total protein and albumin concentrations were estimated by using Biodiagnostic kits, Egypt (CAT.NO. TP2020, cat. no. AB1010), according to the method of Gornall et al. [51] and Doumas et al. [52]. Moreover, plasma samples were collected for antioxidant assays. Estimation of superoxide dismutase (SOD) activity was assayed according to the method of Nishikimi et al. [53]. Glutathione reduced (GSH) was estimated by using a commercial kit (Biodiagnostic Company, Dokki, Giza, Egypt), according to the method of Beutler et al. [54]. Total antioxidant activity (TAO) carried out according to the method of koracevic et al. [55]. Malondialdehyde (MDA) level was assayed using Biodiagnostic kit, Egypt (cat. no. MD2529), according to the method of Satoh [56] and Rubio et al. [57].

### Hematological analysis

#### Complete blood count (CBC)

A portion of retro-orbital blood samples was collected from each animal used for a complete blood count. Blood cell counts (white blood cells, red blood cells, and platelets) were performed with HORIBA Hematology analyzer (model: MICROS 60 OT) (France).

### Sodium dodecyl sulphate polyacrylamide gel electrophoresis (SDS-PAGE)

Protein fractionation was done by using one-dimensional polyacrylamide gel electrophoresis according to the method of Leammli [58].

### Sample preparation

One gram of each sample (liver tissue) was ground in 1 ml homogenate buffer (0.02 M Tris-HCl pH ~ 7.5) using a mortar. The content was transferred to a new Eppendorf tube, centrifuged at 10000 rpm for 10 min at 4 °C, and then the supernatant was kept frozen at − 20°C until required. Protein samples were denatured by boiling in a water bath for 5 min with SDS sample buffer. Then, they were loaded into the wells of gel which was composed of 5% stacking gel and 12% polyacrylamide separating gel next to the molecular weight marker protein. The separated protein on the polyacrylamide gel was stained with Coomassie blue R-250 Andrews [59]. After that, the gel was removed from the distaining solution, placed between two transparent sheets, and was left to air dry. Then, the gel was scanned and analyzed by the Gel-Pro Analyzer program http://meyerinst.com/imaging-software/image-pro/gel/index.htm (Media cybernetics, Georgia, USA).

### Histopathological examinations

Mouse liver tissues were fixed in 10% neutral formalin, then dehydrated, and further embedded with paraffin. Paraffin-embedded liver samples were sectioned to 3-μm-thin slices, which were stained with hematoxylin-eosin (HE) staining and Masson staining according to standard protocols of Oner-lyidogan et al. [60].

### Statistical analysis

Data were evaluated by one-way analysis of variance (ANOVA) by “SPSS” 14.0 for Microsoft Windows, SPSS Inc. and considered statistically significant at a two-sided *p* < 0.05. Numerical data were expressed as mean ± SD.

## Results

### Characterization of curcumin

#### The melting point of curcumin

The prepared curcumin was crystallized from absolute ethanol as a yellow-orange crystalline solid, melting at 180–181 °C which is effective concur with the literature (m.p = 181–182 °C, [42, 61]; and m.p = 178–182 °C, [62]).

#### Infrared spectroscopy (IR) of curcumin

The IR spectrum of curcumin is shown in Fig. 3 along with its data and assignments, which are effective and concur with the literature [42, 63–68].

#### Nuclear magnetic resonance spectroscopy of curcumin

The ^1^H and ^13^C NMR spectra of curcumin are shown in Figs. 4 and 5, respectively. The NMR data of curcumin, whose interpretation confirmed its structure and its effectiveness, concurring with the literature [42, 64, 67, 68].

### Mass spectrometry (MS) of curcumin

The MS analysis has confirmed the structure of curcumin, showing its molecular ion peak at m/z = 368 with a relative intensity of 0.92%.

#### Curcumin and sulfamethoxazole relieved CCl_4_-induced on antioxidants in Swiss mice

**Fig. 6:**
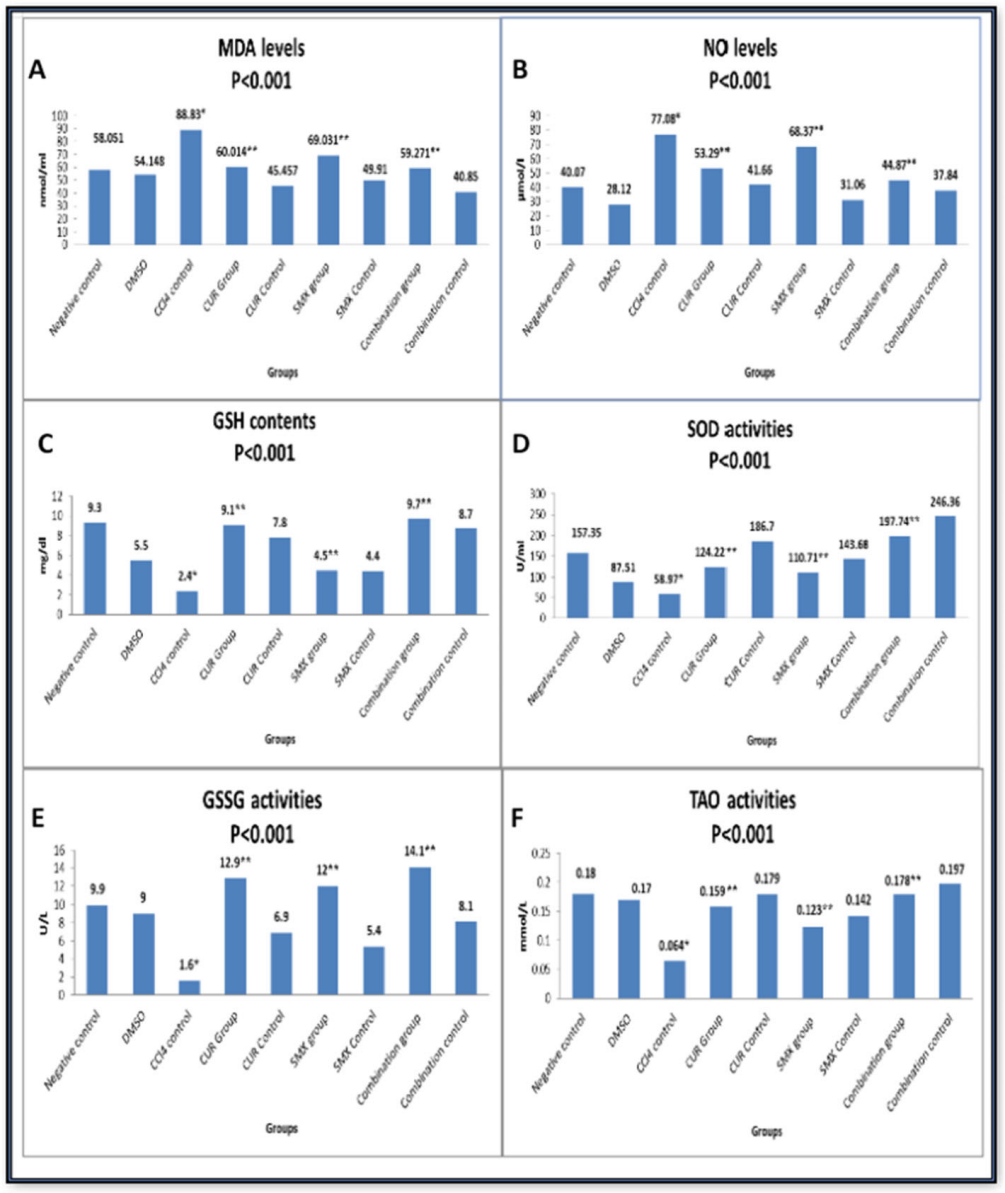
Effect of curcumin and sulfamethoxazole on antioxidants in all studied groups. **a** MDA levels, **b** O levels, **c** GSH contents, **d** SOD activities, **e** GSSG activities, and **f** TAO activities. Results were expressed as mean. (*) Compared with normal control, (**) Compared with CCl_4_ control showed a highly significant difference from control value at (*p* < 0.001)

The mean value of MDA and NO levels were 58.051 + 0.92 nmol/ml and 40.07 ± 0.71 μmol/l, respectively in a group (I, Table 1). The CCL_4_-positive control group (III, Table 1) showed a significant increase in MDA and NO levels to be 88.83 + 0.70 nmol/ml and 77.08 ± 0.57 μmol/l, respectively, (*p* < 0.001) compared to the control group (I, Table 1). Their levels demonstrated a significant decrease in curcumin (IV, Table 1), sulfamethoxazole (VI, Table 1), and their combination group (VIII, Table 1) compared to the CCl_4_-positive control group (III, Table 1) as shown in Fig. 6a, b. On the other hand, SOD, GSH, GSSG, and TAO activities were decreased (*p* < 0.001) from 157.35 + 0.75 U/ml, 9.3 + 0.54 mg/dl, 9.9 + 0.24 U/L, and 0.18 + 0.022 mmol/L in the negative control group (I, Table 1) to 58.97 + 0.65, 2.4 + 0.54, 1.6 + 0.26, 0.064 + 0.011, respectively in CCL_4_ control group (III, Table 1). While their concentration was significantly increased in the curcumin group (IV, Table 1), sulfamethoxazole group (VI, Table 1) and highly increased in the combination group (VIII, Table 1) compared to the CCL_4_-positive control group as shown in Fig. 6c–f, respectively.

#### Curcumin and sulfamethoxazole relieved CCl_4_-induced on liver enzymes in Swiss mice

**Fig. 7:**
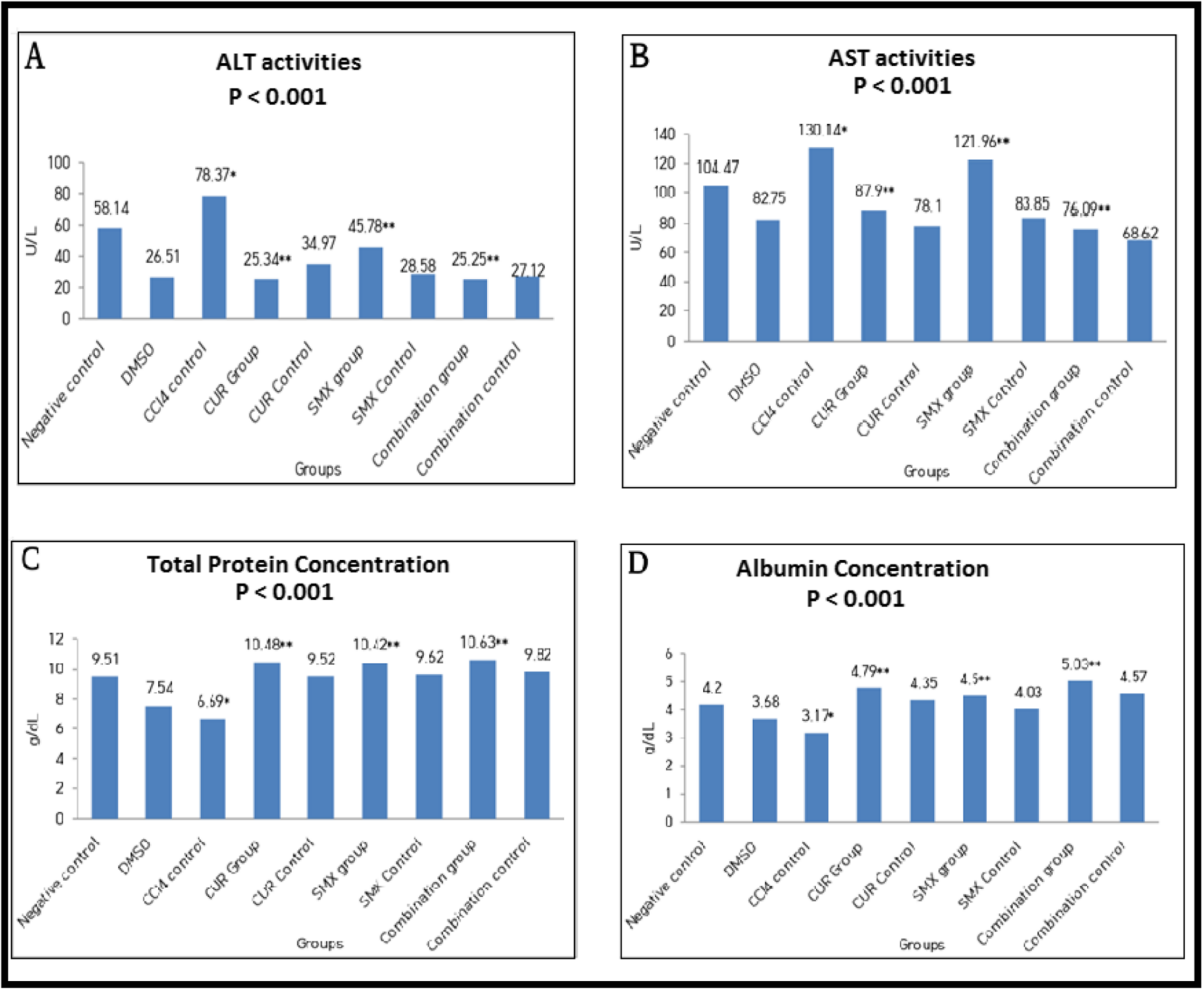
Biochemical markers of hepatic injury (liver function tests). **a** ALT activities, **b** AST activities, **c** total protein concentrations, and **d** albumin concentrations. Results were expressed as mean (*) compared with normal control, (**) compared with CCl_4_ control, and showed a highly significant difference from control value at (***p*** < 0.001)

Measurement of liver enzyme activities demonstrated a significant increase in ALT and AST activities in CCl_4_-positive control group (III, Table 1) to 78.37 + 4.3 and 130.14 + 5.0 U/L; respectively, in comparison to the negative control group (I, Table 1) (*p* < 0.001). These high activities of liver enzymes were significantly reduced by curcumin (IV, Table 1), sulfamethoxazole (VI, Table 1), and their combination (VIII, Table 1) in comparison with CCl_4_-positive control group (III, Table 1), (*p* < 0.001) as in Fig. 7a, b. Also, measurement of total protein and albumin concentration demonstrated a significant decrease in CCl_4_-positive control group (III, Table 1) to 6.69 + 0.24 and 3.17 + 0.28 g/dl, (*p* < 0.001) in comparison with a negative control group (I, Table 1).These concentrations were significantly increased to 10.48 + 0.83 and 4.79+ 0.17, respectively in (CCl_4_ + CUR) group (IV, Table 1), to 10.42 + 0.41 and 4.50 + 0.37 in (CCl_4_ + SMX) group (VI, Table 1) and o 10.63 + 0.36 and 5.03 + 0.15, in (CCl_4_ + CUR + SMX)group (VIII, Table 1), respectively, compared to CCl_4_-positive control group (III, Table 1), (*p* < 0.001) as in Fig. 7c, d.

#### Curcumin and sulfamethoxazole relieved CCl_4_-induced on hematological changes in Swiss mice

**Fig. 8:**
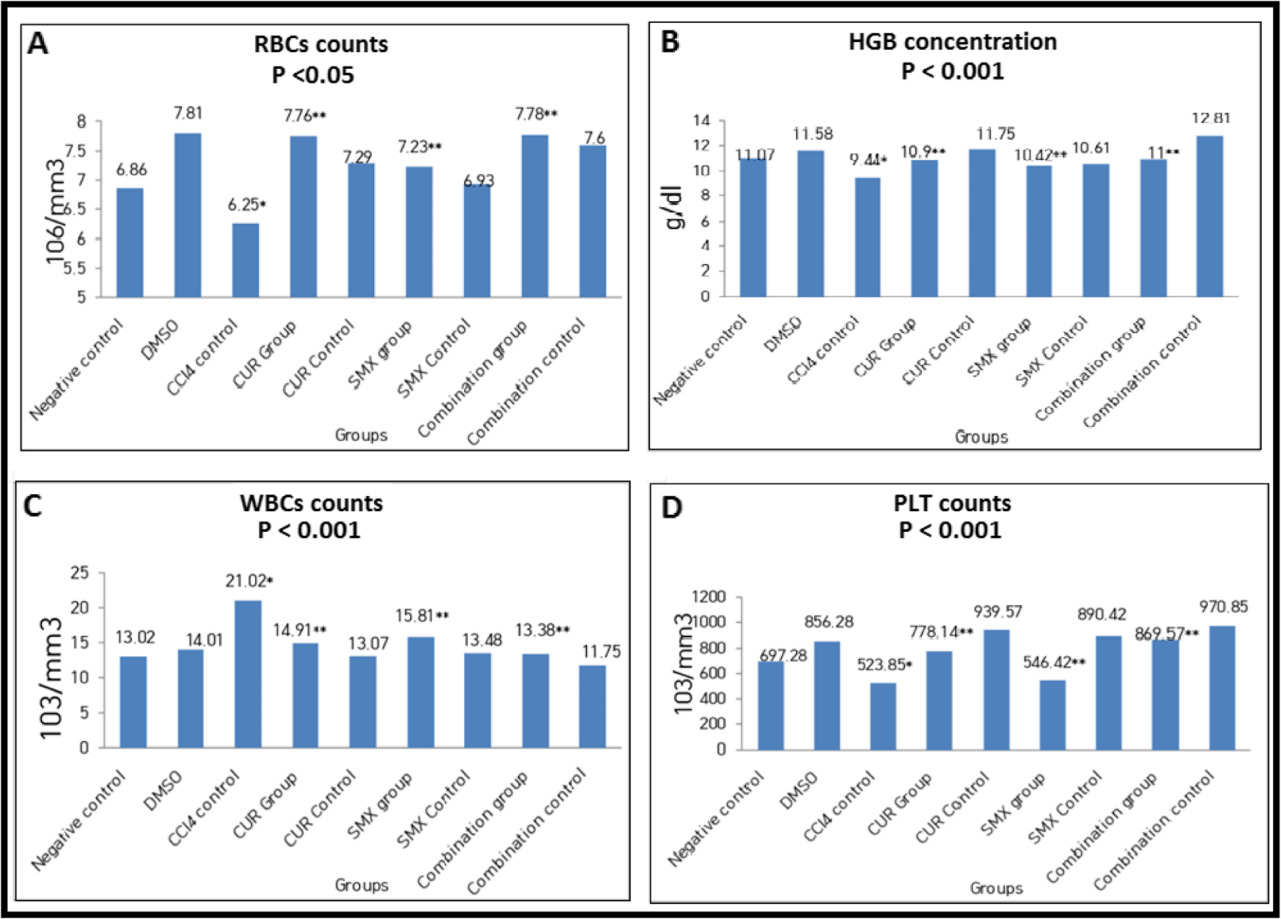
Effect of curcumin and sulfamethoxazole on hematological changes in all studied groups. **a** RBCS counts, **b** HGB concentrations, **c** WBCs counts. **d** PLT counts. Results were expressed as mean (*) compared with normal control, (**) compared with CCl_4_ control, and showed a highly significant difference from control value at (*p* < 0.001) except for RBCs which was at (*p*< 0.05)

Red blood cells (RBCs) count, hematocrit (HCT) value, platelets (PLT) count, and hemoglobin (HGB) concentration were significantly decreased to 6.25 + 0.29 × 10^6^/mm^3^ (*p* < 0.05), 27.30 + 1.27%, 523.85 + 22.57 × 10^3^/mm^3^, and 9.44 + 0.51 g/dl (*p* < 0.001), respectively. While white blood cells (WBCs) count was significantly increased to 21.02 + 2.0 × 10^3^/mm^3^ (*p* < 0.001) in the control CCl_4_-positive control group (III, Table 1) in comparison with the negative control group (I, Table 1). RBCs count, HGB concentration, HCT value, PLT count, and HGB concentration were significantly increased in curcumin group, sulfamethoxazole group, and in combination CUR + SMX group, while WBCs count was significantly decreased in curcumin group (IV, Table 1), sulfamethoxazole group (VI, Table 1), and in combination CUR + SMX group (VIII, Table 1) in comparison with CCL_4_-positive control group (III, Table 1) as in Fig. 8a–d.

#### Sodium dodecyl sulphate polyacrylamide gel electrophoresis (SDS-PAGE) on liver tissue of Swiss mice

**Fig. 9:**
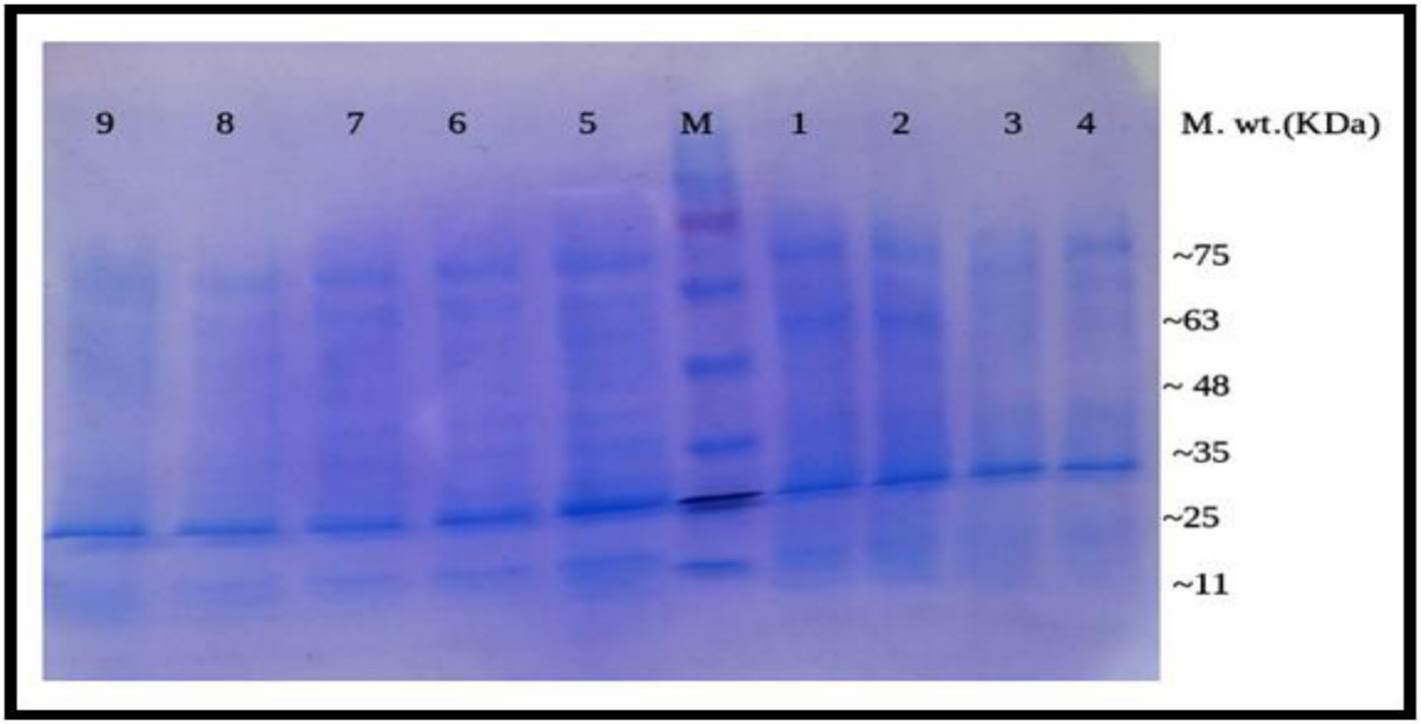
SDS-Polyacrylamide gel of protein pattern of liver tissue samples. M = Marker 11–75 KDa, lanes 1–9: groups 1–9. Lane 1 represents the electrophoretic pattern of CCl_4_ group, lane 2 represents the electrophoretic pattern of sulfamethoxazole group, lane 3 represents the electrophoretic pattern of combination (curcumin + sulfamethoxazole) group, lane 4 represents the electrophoretic pattern of curcumin group, lane 5 represents the electrophoretic pattern of the DMSO control group, lane 6 represent the electrophoretic pattern of combination (curcumin + sulfamethoxazole) control group, lane 7 represents the electrophoretic pattern of sulfamethoxazole control group, lane 8 represents the electrophoretic pattern of curcumin control group, and lane 9 represent the electrophoretic pattern of the normal control group.

The electrophotography showed that the protein band, especially at M.wt nearly 53 KDa, in CCL_4_-positive control group (lane-1) completely disappeared in the normal control group (lane-9), DMSO control group (lane-5), curcumin control group (lane-8), sulfamethoxazole control group (lane-7), and combination (curcumin + sulfamethoxazole) control group (lane-6). However, the band partially disappeared in the curcumin group (lane-4), sulfamethoxazole group (lane-2), and combination (curcumin + sulfamethoxazole) group (lane-3) as in Fig. 9.

**Fig. 10:**
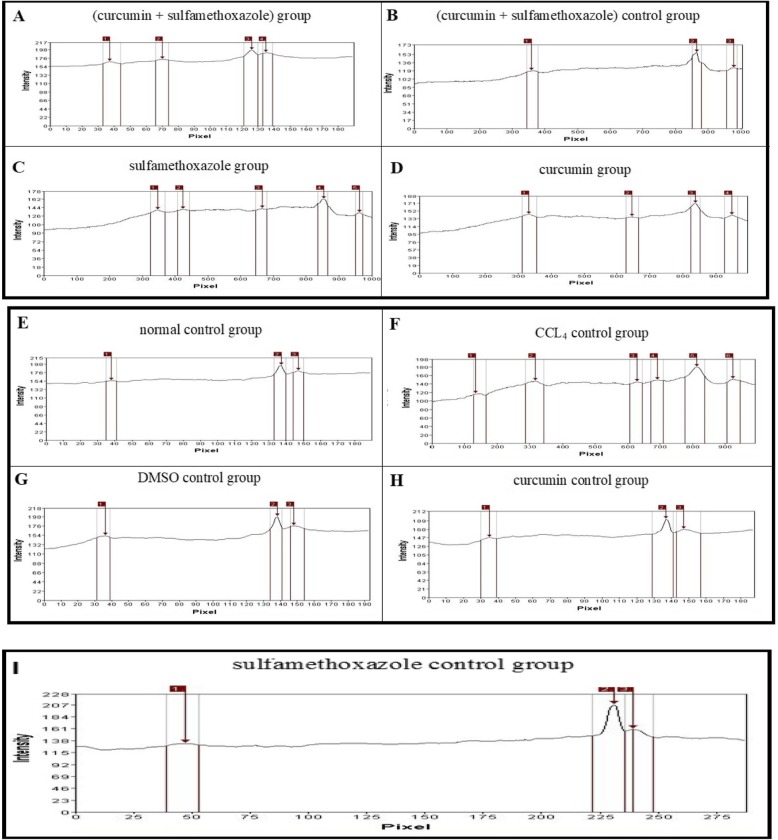
Gel Pro analysis of protein pattern of liver tissue samples by SDS electrophoresis SDS of combination (curcumin + sulfamethoxazole) group (**a**), (curcumin + sulfamethoxazole) control group (**b**), sulfamethoxazole group (**c**), curcumin group (**d**), normal control group (**e**), CCl_4_ control group (**f**), DMSO control group (**g**) curcumin control group (**h**), sulfamethoxazole control group (**i**)

The gel pro analysis demonstrated that six bands in CCL_4_-positive control group, three bands in normal control group, three bands in DMSO control group, three bands in sulfamethoxazole control group, three bands in curcumin control group, three bands in combination (curcumin + sulfamethoxazole) control group, four bands in curcumin group, five bands in sulfamethoxazole group, and four bands in combination (curcumin + sulfamethoxazole) group with molecular weight approximately ranged from 11 to 75 KDa as in Fig. 10a–i

### Histopathological studies

**Fig. 11:**
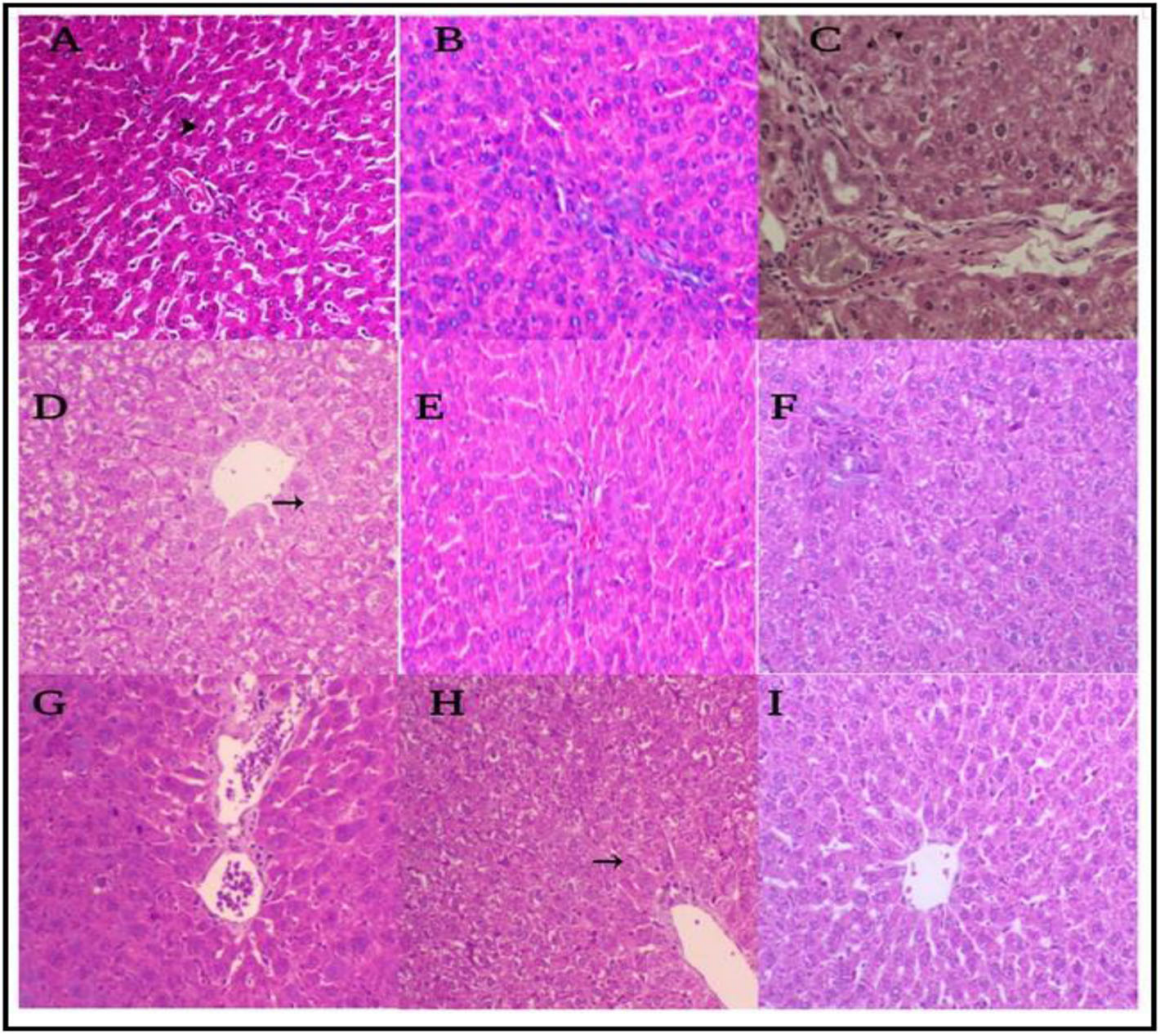
A photomicrograph of the effects of CUR and SMX on CCl_4_-induced liver fibrosis in Swiss mice, H&E, × 200,400. **a** Normal hepatocytes around the portal area. **b** Normal liver structures. **c** Degenerative changes with an increased number of fibroblast and fibrosis. **d** Swelling of hepatocytes within the centrilobular area (arrow) in addition to a moderate degree of vacuolization of hepatocytes within the periportal area. **e** Normal liver structure. **f** Cloudy swelling and vacuolar degeneration of hepatocytes and mononuclear cells infiltration. **g** Normal liver structure. **h** Swelling and mild vacuolation of hepatocytes. **i** Normal liver structure

The histological examination of liver tissues in all studied groups confirmed the biochemical study in all different groups (Fig. 11). The histological examination of the control mice group (negative control) (I, Table 1) showed, the normal hepatocytes around the portal area (arrowhead), H&E, × 200, (Fig. 11a). The DMSO group (II, Table 1) showed normal liver structures, H&E, × 200, (Fig. 11b). However, the CCl_4_ group (III, Table 1) had degenerative changes with an increase number of fibroblasts and fibrosis, H&E X400, (Fig. 11c). Meanwhile, the curcumin-treated group (IV, Table 1) showed cell swelling of hepatocytes within the centrilobular area (arrow) besides a moderate degree of vacuolation of hepatocytes within the periportal area, H&E, × 200, (Fig. 11d). While the curcumin control group (V, Table 1) showed normal liver structures, H&E, × 200, (Fig. 11e). the sulfamethoxazole treated group (VI, Table 1) showed cloudy swelling, vacuolar degeneration of hepatocytes, and mononuclear cells infiltration, H&E, × 200 (Fig. 11f), while the sulfamethoxazole control group (VII, Table 1) showed normal liver structures, H&E, × 200, (Fig. 11G). The combined CUR + SMX group (VIII, Table 1) showed cell swelling and mild vacuolization of hepatocytes, H&E, × 200, (Fig. 11h), while, the combined CUR + SMX control group (IX, Table 1) showed normal liver structures, H&E, × 200, (Fig. 11i).

## Discussion

Curcumin (CUR) is a widely examined natural compound that has demonstrated imposing in vitro therapeutic potential. Disregarding  the way that the experimental efficacy of natural curcumin is frail due to its low bioavailability and high metabolism in the gastrointestinal tract because of its poor solubility in water and quick in liver and intestinal digestion which add to its fast excretion, researchers have focused on ameliorative bioavailability in the most recent decade [69, 70]. Sulfonamides are the central and historically most prominent class of carbonic anhydrase inhibitors, being discovered in 1940, with many representatives in clinical use for decades [71]. Thus, we used sulfamethoxazole with curcumin in combination group (VIII, Table 1) to circumvent the problem of rapid metabolism and to improve its pharmacokinetics profile. The purpose of this study is to investigate the effect of each of curcumin (CUR) and sulfamethoxazole (SMX) either separate or together (CUR + SMX) on biochemical, hematological, and histological alternations associated with carbon tetrachloride (CCl4)-induced liver fibrosis in mice, and to explain the underlying mechanism. The CCl_4_ is a causative agent of numerous disarranges through its capabilityof causing oxidative stress and inflammation. CCl_4_ uses, through enzymes of the CYP 450 system, producing hepatotoxic metabolites for example, trichloromethyl radicals and trichloroperoxyl radicals. These metabolites cause hepatotoxicity through induction of lipid peroxidation that all together gives malondialdehyde (MDA) products that lead to hepatic injury [72]. According to our present data, CCl_4_ caused changes of several biomarkers which proved its hepatotoxic effects such as an increase in aminotransferases liver enzymes (ALT, AST), MDA, and NO, with a decrease in SOD, GSSG, TAO, GSH, total protein, and albumin compared to the negative control group (I, Table 1).

Oxidative stress is the most widely recognized  instrument in organ injury. Oxygen is the primary particle for all cells for the creation of ATP, but it might, likewise can change into harmful species, for example reactive oxygen species. During aerobic respiration, the creation of free radicals could could bring about aging and cell death. Oxygen molecules are reduced by mitochondria to create superoxide ions or hydrogen peroxide (H_2_O_2_). The superoxide and peroxide further respond with metal ions and produce hydroxyl radicals, which react with cell components including DNA and proteins. Polyphenolic curcumin is essentially appended to its antioxidant properties. CUR is seen as ten times more antioxidants than vitamin E [73]. CUR causes an increase in GSH concentration and activity of glutathione peroxidase and SOD. The glutathione and glutathione peroxidase act synergistically to scavenge free radicals. SOD scavenges superoxide radicals by changing over superoxide free radicals into H_2_O_2_ while staying away from the development of hydroxyl radicals. Thus, the H_2_O_2_ formed is expelled by the job of catalase or glutathione peroxidase. In this manner, SOD protects against free radical damage [74]. CUR has antioxidant properties that protect against CCl_4_-induced hepatic damage. It can repair all biomarkers against hepatic damage, i.e., decreasing level of ALT and AST, suppressing lipid peroxidation, decreasing MDA level, recovery of redox balance, and increasing level of reduced GSH [75].

Our results revealed that curcumin-treated group (IV, Table 1) had an increase in SOD, GSSG, TAO, GSH, total protein, and albumin, while it had a decrease in ALT, AST, MDA, and NO in comparison to CCl_4_ group (III, Table 1) which agreed with Zhao et al. [76], who reported that there was an increase in serum biomarkers of ALT, AST, and a decrease in the levels of albumin and the total protein , after treatment with CUR [76]. Indicators in liver disease, ALT and AST are hepatocyte cytosolic enzymes. An increase in levels of ALT and AST usually confirm liver injury. Albumin and total protein levels tend to be reduced in chronic liver injury because of the impaired ability of liver cells to synthesize proteins [76, 77]. Peng et al. [78] showed that curcumin pre-treatment at the doses of 50, 100, and 200 mg/kg markedly alleviated the expansion of MDA level brought about by CCl_4_ and it can up-manage the activities of SOD and levels of GSH. Momeni and Eskandari [79] showed that the use of curcumin alone significantly increased the total antioxidant capacity of serum in comparison with the control. Its direct antioxidant activity, CUR may enhance the synthesis of glutathione and improve the antioxidant defense system, a significant increase in the serum concentration of MDA. Curcumin affected the levels of peripheral blood parameters through increasing HGB, RBC_s_, PLT, and decreasing WBCs, which agreed with the results of Yin et al. [80]; however, the combination between CUR and SMX group (VIII, Table 1) had a significant increase in HGB, RBCs, and PLT and a significant decrease in WBCs, so the combination was more effective than curcumin alone. Sulfamethoxazole-treated group (VI, Table 1) caused an increase in SOD, GSSG, GSH, total protein, and albumin but caused a decreased in ALT, AST, MDA, and NO compared to the CCL_4_ group (III, Table 1) which was in agreement with Sahyon et al. [47]. Gupta et al. [81] reported that (sulfamethoxazole + selenium) treatment resulted in an increase in the activities of SOD, but caused a decrease (*p* < 0.001) in the levels of GSH when compared to diethylnitrosamine-treated animals and showed an increase in the levels of MDA. Also, Bottari et al. [40] reported that the treatments with SMX + trimethoprim, resveratrol, and inclusion complex in free forms or co-administered were able to reduce the total oxidation status and oxidation protein products levels in hepatic tissue of infected animals. In comparison, our results showed that curcumin and sulfamethoxazole combination have significantly, decreased MDA and NO levels, and increased SOD, GSSG, TAO, and GSH levels compared to the corresponding levels of the CCl_4_ control group.

The present data demonstrated an increase in protein synthesis in the CCl_4_ group with a corresponding decrease in the curcumin and/or sulfamethoxazole treated groups. This agreed with Thaloor et al. [82], who reported that the synthesis of proteins (including 60, 36, and 30.7 kDa proteins) was inhibited in the presence of 25 μM of curcumin. In a comparable study, Qin et al. [83] showed that the protein levels were significantly decreased in the CUR-treated groups. This, again, is in concurrence with our results.

The histological examination of liver tissues showed that the liver of the control mice exhibited normal lobular architecture around the portal area. Treatment with CCl_4_ alone caused an increased number of fibroblasts and fibrosis. This agreed with Ahmad et al. [84] who reported that CCl_4_ treated group showed heavy cell infiltration, across the board vacuolated cytoplasm, darkly stained pyknotic and peripheral placed nuclei. Our results, also, agreed with Zhaoa et al. [21] which showed that the liver in CCl_4_ treated mice have a large number of hepatocytes necrosis, leukocytes infiltration, and damaged lobule structure and the liver in CUR treated mice have reduce leukocytes infiltration and reduced hepatocytes necrosis compared to the mice of the CCl_4_-positive control group, which agreed with our CUR treated group and showed cell swelling of hepatocytes within the centrilobular area (arrow), in addition to moderate degree of vacuolation of hepatocytes within the periportal area. The liver of the mice, in the curcumin and sulfamethoxazole combination group (CUR + SMX), showed cell swelling and mild vacuolation of hepatocytes. While the mice group that was treated with sulfamethoxazole (SMX) alone showed cloudy swelling, vacuolar degeneration of hepatocytes, and mononuclear cells infiltration. The above-mentioned results, clearly demonstrated the highly significant effect of curcumin and/or sulfamethoxazole against the harmful effect of CCl_4_ in dealing with Swiss mice group. However, further studies with different dosages and/or concentrations of curcumin and sulfamethoxazole will be done.

## Conclusions

Treatment with curcumin and sulfamethoxazole either separately or together can reduce CCl_4_ hepatotoxicity in mice. Each of curcumin and sulfamethoxazole could be considered a promising candidate in the prevention and treatment of liver fibrosis.

## Data Availability

Authors declare that all generated and analyzed data are included in the article.

## Abbreviations

ALT: Alanine transaminase
AST: Aspartate transaminase
br: Broad
CCl_4_: Carbon tetrachloride
CUR: Curcumin
DMSO: Dimethyl sulfoxide
d: Doublet
EI: Electron impact
ECM: Extracellular matrix
GSSG: Glutathione reductase
HCT: Hematocrit value
HE: Hematoxylin-eosin
HGB: Hemoglobin concentration
HSC: Hepatic stellate cells
IR: Infrared
MDA: Malondialdehyde
MS: Mass spectral
NO: Nitric oxide
NMR: Nuclear magnetic resonance
PLT: Platelets count
KBr: Potassium bromide
RBCs: Red blood cells count
GSH: Reduced glutathione
s: Singlet
SDS-PAGE: Sodium dodecyl sulphate polyacrylamide gel electrophoresis
SMX: Sulfamethoxazole
SOD: Superoxide dismutase
TMS: Tetramethylsilane
m.p.: The melting point
TAO: Total antioxidant activity
